# Coping Mechanisms: Exploring Strategies Utilized by Japanese Healthcare Workers to Reduce Stress and Improve Mental Health during the COVID-19 Pandemic

**DOI:** 10.3390/ijerph18010131

**Published:** 2020-12-27

**Authors:** Masatoshi Tahara, Yuki Mashizume, Kayoko Takahashi

**Affiliations:** 1 Graduate School of Medical Sciences, Kitasato University, 1-15-1, Kitasato, Minami-ku, Sagamihara, Kanagawa 252-0373, Japan; dm18026@st.kitasato-u.ac.jp (Y.M.); kayo.ot@kitasato-u.ac.jp (K.T.); 2 Department of Rehabilitation Therapist, Saiseikai Higashikanagawa Rehabilitation Hospital, 1-13-10, Nishikanagawa, Kanagawa-ku, Yokohama, Kanagawa 221-0822, Japan; 3 Department of Occupational Therapy, School of Allied Health Sciences, Kitasato University, 1-15-1, Kitasato, Minami-ku, Sagamihara, Kanagawa 252-0373, Japan

**Keywords:** COVID-19, coping strategy, healthcare worker, mental health, stress coping

## Abstract

The COVID-19 pandemic is a major problem affecting the mental health of millions of people, including healthcare workers. In this study, we analyzed risk factors and coping mechanisms that could reduce the risk of poor mental health among healthcare workers during the COVID-19 pandemic in Japan. A cross-sectional survey was conducted for 7 days from 30 April 2020 using a web-based questionnaire. The survey assessed various outcome measures, including the General Health Questionnaire-12 (GHQ-12), health status, satisfaction with daily life activities, work, leisure, and new activities, and anxiety over COVID-19. Data from 661 participants were analyzed, and 440 participants (66.6%) showed poor mental health (GHQ-12 ≥ 4). Also, our result showed that female gender, lower levels of communication with friends, and high anxiety were associated with poorer mental health. In contrast, good health status, high work satisfaction, and high satisfaction from new activities were associated with buffering mental health problem. Most participants chose an escape-avoidance coping strategy, and participants with worse mental health were more likely to adopt seeking social support as a coping strategy. These results may support healthcare workers to cope with mental health problems associated with the COVID-19 pandemic.

## 1. Introduction

In December 2019, a new type of viral pneumonia began to spread in Wuhan, China [1], and in January 2020, the disease began to extend across Japan as well [2]. The novel virus, named ‘severe acute respiratory syndrome coronavirus 2’ (SARS-CoV-2), quickly spread throughout the world, and on 11 March 2020, the World Health Organization (WHO) declared coronavirus disease 2019 (COVID-19) a pandemic [3]. The number of confirmed infected people in Japan surged to more than 2500 by late March [2], and the number of potentially infected people was expected to be higher. Under these circumstances, the Japanese government issued an emergency declaration for all prefectures in Japan on 7 April 2020, and designated 13 prefectures, including Tokyo and Osaka, as ‘special alert’ regions [4]. With this declaration, ‘social distancing’ was emphasized, and people were requested to reduce contact with other individuals by 80% and limit their nonessential and non-urgent outings.

Healthcare workers have been especially affected by this pandemic, which has led to serious mental health problems [5,6]. With the spread of COVID-19, the healthcare system in Japan (as in other countries) became strained, with deterioration in conditions because of a lack of supplies, hospital beds, and personnel, as well as crowded hospitals caring for many patients with confirmed or possible COVID-19 infection. Deterioration of people’s mental health has been a problem in past pandemics of widespread infectious diseases [7,8,9,10], but COVID-19 is a worldwide epidemic, and lockdowns and other movement restrictions have been taken in many countries. Even in Japan, such restrictions and tight medical care are unprecedented, and there is concern that mental health may deteriorate [11,12]. These conditions produced anxiety among healthcare workers because of fears of becoming infected themselves [13].

Consequently, stress and psychological problems among healthcare workers working under such harsh conditions have precipitated serious health conditions, including insomnia, depression, and anxiety [5,6]. In addition to workplace-associated stresses, restrictions impacting daily life, such as limitations on public outings, have reduced opportunities for medical personnel to release stress, further worsening their mental status [14]. Therefore, determining risk and resilience factors associated with mental health problems secondary to COVID-19 [15] is crucial to provide support to individuals at risk and enhance resilience factors.

Similarly, it is important to identify stress coping strategies that help healthcare workers adjust to the highly stressful circumstances associated with COVID-19. Coping is defined as a person’s constantly changing cognitive and behavioral efforts to manage specific external and/or internal demands considered taxing or exceeding a person’s resources [16]. Stress coping strategies during the 2002 SARS epidemic were shown to improve psychological symptoms, life satisfaction, and general health [17]. In addition, previous studies have reported that in past pandemics, healthcare workers were able to cope with the predicament by controlling their emotions and adapting to the situation [18]. Hence, using coping strategies in stressful situations may prevent a mental health crisis, and proposing strategies healthcare workers can use to cope with stress will be an essential element in countering the negative effects of COVID-19.

In this study, we conducted a cross-sectional analysis using a web-based survey to investigate risk and resilience factors associated with the mental status of healthcare workers during the COVID-19 pandemic and to identify stress coping strategies for reducing the risk of poor mental health among this population.

## 2. Materials and Methods

### 2.1. Design and Participants

A cross-sectional survey of healthcare workers was conducted using a web-based questionnaire (Google Form^®^ by Google LLC, Los Angeles, CA, USA) from 30 April 2020 to 6 May 2020, after implementation of social distancing restrictions in Japan. Since the clinical practice in Japan was tight and confusing, a survey using snowball sampling was adopted in consideration of the reality.

The initial participants were 867 graduates of the occupational therapy program of Kitasato University, and further participants were recruited using the snow-ball method. The inclusion criteria were occupation as a physician, nurse, physical therapist, occupational therapist, or speech and language pathologist, and working full-time. 

### 2.2. Survey Items

Survey items included demographic information, including age, gender, marital status, number of children (aged ≤ 18 years), number of people in the household, region of residence, occupation, employment status, clinical phase of workplace (for example, acute hospital, subacute hospital, chronic hospital), years of clinical experience, amount of communication with friends and family, and financial status. Region of residence was divided into alert region (13 special alert prefectures) and non-alert region (remaining 32 prefectures). Workplace clinical phase was categorized as acute, subacute, or chronic. Amount of communication with family and friends, as well as financial status, was rated on a three-point Likert scale: ‘same as usual’, ‘less than usual’, or ‘more than usual’, compared with pre-COVID-19 pandemic.

Mental health status was measured using the Japanese version of the General Health Questionnaire-12 (GHQ-12), which includes 12 questions regarding mental health status, with four options for each question, and higher scores represent worse mental health. In the four options for each question, 6 items of negative expressions are arranged in the order of “Not at all”, “Less than usual”, “Same as usual”, “More than usual”, and 6 items of positive expressions are “More than usual”. Answers are arranged in the order of, “Same as usual”, “Less than usual”, “Not at all”.

The reliability and validity of the Japanese version of the GHQ-12 have already been reported [19]. The GHQ-12 has different scoring methods: we used the bimodal method (0-0-1-1) in this study [20]. Based on the cut-off value in previous studies, a GHQ-12 score ≥ 4 was used as the criterion for severe mental health problems [21,22]. Health status and anxiety over COVID-19 were investigated using a visual analog scale, rated from 1 (‘not at all anxious’ or ‘not at all healthy’) to 10 (‘very anxious’ or ‘very healthy’).

Effects on daily life of restraints related to COVID-19 infection control were investigated using our original scale based on the Canadian Occupational Performance Measure (COPM) [23,24]. COPM uses a ten-point Likert scale to evaluate satisfaction and performance of daily life activities in the areas of self-care, productivity, and leisure. In addition, COPM is translated and validated in Japanese [25]. In this study, we investigated four aspects of daily life satisfaction: satisfaction with leisure, satisfaction with job, satisfaction with daily life activities, and satisfaction with new activities started since social distancing began. Each item was rated on a 10-point Likert scale, from 1 (‘not satisfied at all’) to 10 (‘very satisfied’). For satisfaction with new activities, ‘no new activities’ was included in the ‘1′ rating.

Coping strategies as behavioral efforts to manage stress were assessed using an open-ended question: “Please write down what you do to calm yourself down or release stress”. We used this approach to capture the potentially wide variety of strategies incorporated by participants.

### 2.3. Data Analysis

For statistical analyses, SPSS software (version 25, IBM Corp.) was used to compare gender, workplace phase, and region of residence subgroups using the Mann–Whitney and Kruskal–Wallis tests. We determined risk factors for mental health problems using the logistic regression analysis with GHQ-12 as the dependent variable.

To evaluate coping strategies, comments on behavioral efforts to manage stress were deductively categorized according to strategies used in previous research [26]: confrontive coping, distancing, self-control, seeking social support, accepting responsibility, escape-avoidance, planful problem-solving, and positive reappraisal. Confrontive coping describes aggressive efforts to alter situations and involves a degree of hostility and risk-taking. Distancing describes efforts to detach oneself. Self-control describes efforts to regulate one’s own feelings and actions. Seeking social support describes efforts to seek informational, tangible, or emotional support. Accepting responsibility involves acknowledging one’s role in a problem, while concomitantly trying to improve the situation. Escape-avoidance describes wishful thinking and behavioral efforts to escape or avoid problems. Planful problem-solving describes deliberate problem-focused efforts to alter situations, coupled with analytic approaches to solving problems. Positive reappraisal describes efforts to create positive meaning by focusing on personal growth. We calculated the total number of behavioral efforts and percentages for each coping strategy to understand characteristics of participants with and without mental health problems (GHQ-12 ≥ 4).

## 3. Results

Responses were received from 801 individuals during the 7-day period from 30 April 2020, to 6 May 2020. Data from 661 participants (82.5%) who met the inclusion criteria were analyzed.

### 3.1. Demographics

Table 1 summarizes demographic data. The participants included 354 women (53.6%). The age distribution was as follows: 21–25 years, 164 participants (24.8%), 26–30 years, 201 participants (30.4%), and 31–40 years, 198 participants (30.0%). A total of 281 participants (42.5%) were married, and 200 (30.3%) lived with ≥1 child. In terms of living status, 195 (29.5%) lived alone, 145 (21.9%) lived with 1 other person, and 321 (48.6%) lived with ≥3 other people. A total of 506 participants (76.6%) lived in the alert region and 155 (23.4%) lived in the non-alert region.

Participants included 507 occupational therapists (76.7%), 122 physiotherapists (18.5%), 16 speech and language pathologists (2.4%), 8 physicians (1.2%), and 8 nurses (1.2%). Their workplaces were almost equally distributed among acute, subacute, and chronic phases: 219 (33.1%), 215 (32.5%), and 227 (34.3%), respectively. Regarding clinical experience, 255 (38.6%) participants had 1–5 years of experience, 197 (29.8%) had 6–10 years, and 163 (24.7%) had 11–20 years. Regarding their financial situation, 505 participants (76.4%) answered ‘no change’, and 66 (10.0%) answered ‘worse than usual’. For amount of communication with family, 324 participants (49.0%) answered ‘same as usual’, and 114 (17.2%) responded ‘less than usual’. Conversely, 192 participants (29.0%) answered ‘same as usual’ and 403 (61.0%) responded ‘less than usual’ for amount of communication with friends. With regards to the structure of the population of occupational therapists and physiotherapists in Japan, who account for a large part of the participants in this study, the percentage of male is 53.7% and 46.3% for female. As for age distribution, the portion was as follows: 16.9% for 21–25 years, 25.6% for 26–30 years, 36.1% for 31–40 years, and 21.5% for over 40 years. Moreover, 53.0% of occupational therapists and physiotherapists in Japan live in the alert region [27,28].

### 3.2. Severity of Mental Health Problems and Associated Factors

The GHQ-12 score was ≥4 in 440 participants (66.6%). Median score for anxiety over COVID-19 was 9 (interquartile range (IQR): 7–10). Other median scores were 7 (IQR: 5–9) for health status, 2 (IQR: 1–3) for satisfaction with leisure, 6 (IQR: 3–8) for satisfaction with work, 4 (IQR: 3–7) for satisfaction with daily life activities, and 6 (IQR: 3–8) for satisfaction with new activities started since social distancing began.

Mental health measurements were analyzed for gender, workplace clinical phase, and place of residence subgroups (Table 2). For intergroup comparisons according to gender, women had significantly higher GHQ-12 score and anxiety over COVID-19 and less satisfaction with leisure, compared with men. For comparisons according to workplace phase, satisfaction with work differed significantly between phases (*p* = 0.01). No significant differences were observed between place of residence subgroups.

Table 3 shows demographic characteristics and mental health measurements for normal and severe mental health problems groups. The severe mental health problems group (GHQ-12 score ≥ 4) exhibited these characteristics: 263 (59.8%) were women, 147 (33.4%) lived alone, 92 (20.9%) lived with 1 other person, and 201 (45.7%) lived with ≥2 other people, 328 (74.5%) answered ‘no change’ and 48 (10.9%) responded ‘worse than usual’ for financial situation, and 97 (22.0%) answered ‘same as usual’ and 302 (68.6%) responded ‘less than usual’ for communication with friends.

### 3.3. Resilience Factors for Mental Health Outcomes

After adjusting for confounding variables in the logistic regression analysis, women had an increased risk of mental health problems (odds ratio (OR), 1.83; 95% confidence interval (CI), 1.25–2.7; *p* = 0.02). Living with 1 other person (OR, 0.47; 95% CI, 0.27–0.80; *p* = 0.006) and living with ≥2 other people (OR, 0.52; 95% CI, 0.33–0.82; *p* = 0.005) were associated with a lower risk of mental health problems, compared with living alone. Less communication than usual with friends (OR, 2.29; 95% CI, 1.51–3.46; *p* < 0.001) and high anxiety over COVID-19 (OR, 1.20; 95% CI, 1.08–1.34; *p* = 0.001) were associated with an increased risk of mental health problems. Conversely, high health status (OR, 0.76; 95% CI, 0.69–0.83; *p* < 0.001), high work satisfaction (OR, 0.82; 95% CI, 0.75–0.90; *p* < 0.001), and high satisfaction with new activities started since social distancing began (OR, 0.92; 95% CI, 0.86–0.99; *p* = 0.01) were associated with a reduced risk of mental health problems (Table 4).

### 3.4. Coping Strategy

**Figure 1:**
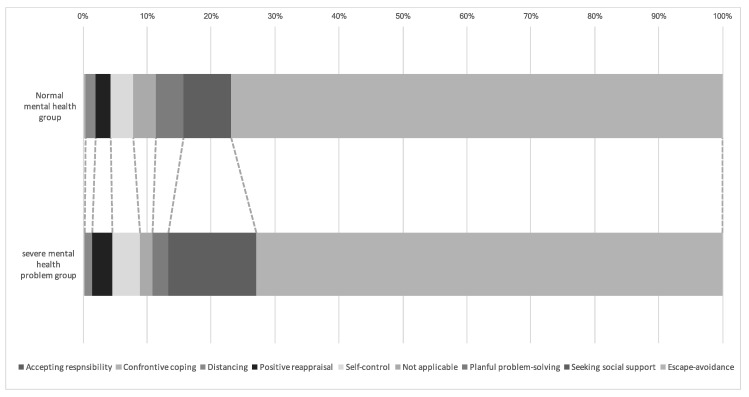
Coping strategies classified using descriptive data according to differences in mental health status.

At least one coping strategy was used by 153 of 221 participants (69.2%) in the normal mental health group and 309 of 440 participants (70.2%) in the severe mental health problems group (Figure 1). Escape-avoidance was used by 196 participants in the normal mental health group (76.9%) and 401 in the severe mental health problems group (72.9%). Seeking social support was a coping strategy for 19 participants in the normal mental health group (7.5%) and 76 in the severe mental health problems group (13.8%).

## 4. Discussion

First, regarding the validity of demographic data, Occupational therapists (76.7%) and physiotherapists (18.5%) had a large proportion of participants. The percentage of occupational therapists aged 21 to 30 in Japan is 42.0%, and the percentage of physiotherapists aged 21 to 30 is 42.6% [27,28]. Therefore, the result of this study is that the ratio of 21 to 30 years old is 55.4%, which does not deviate significantly from the age distribution of general healthcare workers.

Based on GHQ-12 scores, 66.6% of study participants had mental health problems. Regarding a previous study [22], in pre-COVID-19, the prevalence of “mental health problems (GHQ-12 ≥ 4)” in the general Japanese population was 30–40%. A meta-analysis reported prevalence rates of 23.2% for anxiety and 22.8% for depression among healthcare workers during the COVID-19 pandemic [6]. Another study reported that stress levels among medical staff during the pandemic were higher than usual [29]. In addition, previous studies on the mental health of the general public and healthcare workers in the COVID-19 pandemic/epidemic have been reported [7,30,31]. Our results indicate that in Japan, as in other countries, substantial mental health problems have developed among healthcare workers since implementing COVID-19 infection control measures [5,32].

Rates of mental health problems were previously reported to be higher in healthcare workers than in the general population [31]. Approximately 40.4% of the general population was reported to have psychological problems resulting from stress associated with COVID-19 [33], which was lower than the 66.6% rate in our study participants. This disparity could be at least partly attributed to the effects of stigmatization and discrimination towards medical professionals [34,35] as well as anxiety over the higher risk of infection [36]. Our findings confirm that healthcare professionals are experiencing serious mental health issues during the COVID-19 pandemic, emphasizing the need for action.

Our results showed a gender gap, with females having a risk of severe mental health problems. In general, prevalence rates of anxiety and depression are higher in females [37], and our results may reflect this general observation. Increased risk of severe mental health problems was associated with factors related to loneliness, including living alone and reduced communication with friends. Loneliness has been linked to numerous mental disorders, such as depression and sleep disorders, and continuing social relationships is important for maintaining a sense of well-being [38,39]. Therefore, healthcare workers living alone and with less communication, especially when female, should be considered at higher risk for mental health problems. Since there is a limit to clearly showing the causal relationship of each risk factor from the results of this study, it is necessary to consider it when providing support by referring to this result.

Our study also identified several resilience factors, including high health status and high job satisfaction. Previous studies reported negative correlations between job satisfaction and mental or psychosocial problems [40], while others reported positive relationships between high resilience and job satisfaction or general health [41]. Therefore, healthcare workers who are satisfied with their jobs and find their work worthwhile may be less likely to develop mental health problems. High satisfaction with new activities started since social distancing began was another identified resilience factor. During self-quarantine and restriction, outdoor leisure activities that alleviate psychological distress [42] may be difficult, and poor lifestyle choices and health behaviors may promote depression [43]. Conversely, satisfying new activities may prevent mental health deterioration.

Resilience is described as a tool or skill to change, balance, or control oneself in an unfavorable environment [44]. Although it is difficult or impossible to change living style or gender, which were risk factors of poor mental health in this study, it may be possible to avert mental health deterioration by increasing resilience. Resilience factors are positive coping mechanisms for COVID-19-related changes in work and life routines [45], which are powerful tools for enhancing the ability to cope with the mental health crisis associated with COVID-19. Job satisfaction was a resilience factor, but because of stigmatization and discrimination towards healthcare workers [35,36], individuals may have difficulty being satisfied with their work, potentially worsening their mental state. Providing emotional support to healthcare workers may help maintain psychological well-being.

Satisfaction with new activities started since implementing social distancing was another resilience factor observed in our study. Most previous studies focused on reducing risk factors, such as anxiety and poor health status [46], but our findings revealed that healthcare workers have been adopting proactive strategies to reduce stress and increase resilience. This is a novel finding, which provides new insight into preventing mental health problems. To minimize social isolation and loneliness, which were identified as risk factors for poor mental health [47], participants incorporated new activities, such as online communication, into their routines. Many participants also engaged in health management activities. Physical exercise helps maintain good health [48] while avoiding infection, and engaging in productive activities, such as arts and crafts, has demonstrated therapeutic effects [49]. To increase resilience, appropriate work–life balance must be maintained. Thus, incorporating communication, health management, and productive activities into daily routines may help prevent COVID-19-related mental health problems.

Over 70% of study participants adopted the escape-avoidance strategy for coping with stress. Previous studies found that mental health effects vary depending on the selected stress coping strategy [50], and the escape-avoidance strategy could worsen mental health [50] and increase psychological distress and depression [51]. However, in uncontrollable situations, escape-avoidance coping strategies are more likely to be adopted [52,53]. As our participants faced restrictions even in their daily lives to prevent the spread of COVID-19, this strong external pressure may have forced them to adopt the escape-avoidance coping strategy. That is, an evasive strategy may have been the best way to maintain mental health. However, starting satisfying activities may be a more appropriate strategy for preventing mental health deterioration.

Seeking social support as a coping strategy was adopted more frequently by participants with poor mental health than those with good mental health. Seeking social support includes not only seeking emotional support but also informational and tangible support to ease anxiety. However, when infectious diseases are still emerging, it is difficult to obtain accurate information, and using the internet for this purpose may be counterproductive. Prolonged internet usage may increase depression and psychological distress [54] and using the internet as an avoidance coping strategy may increase anxiety [55].

One limitation of this study is possible selection bias regarding participants. In previous studies [56,57], surveys using snowball sampling and SNS have been conducted, but it is difficult to sufficiently eliminate selection bias. Occupational therapists accounted for 76.7% of participants, which does not reflect their percentage of all healthcare workers. Our study may have also been affected by regional bias, as 76.0% of participants lived in the alert region, which included urban areas; thus, workers in rural areas were underrepresented. Furthermore, the study was limited to healthcare professionals, so the results do not reflect trends in the general public. Use of a web-based survey made it difficult to eliminate duplicate answers, and because the survey was widely distributed via social networks, we could not determine whether the response rate appropriately reflected the population. Furthermore, the study was limited to 7 days and therefore did not assess long-term mental health problems. Additional longitudinal studies would be useful to investigate mental health status changes over time.

## 5. Conclusions

In conclusion, over half of healthcare workers surveyed reported mental health problems since initiating infection control restrictions because of COVID-19 in Japan, with female gender and loneliness identified as risk factors. Being satisfied with work and starting a new activity were possible ways to counter mental health deterioration. While escape-avoidance coping was often adopted, excessive information seeking may adversely affect mental health, and adopting satisfying activities may be a more appropriate way to prevent mental health deterioration. Our results not only provide insight into coping strategies that can be practiced by healthcare workers during the COVID-19 era, but they may also act as a reference for addressing mental health crises among healthcare workers during the spread of any highly infectious disease.

## Figures and Tables

**Table 1:** Demographic characteristics of study participants (*N* = 661).

Characteristics	Number (%)
Gender	
Men	307 (46.4)
Women	354 (53.6)
Age, years	
21–25	164 (24.8)
26–30	201 (30.4)
31–40	198 (30.0)
>40	98 (14.8)
Marital status	
Single	380 (57.5)
Married	281 (42.5)
Occupation	
Physician	8 (1.2)
Nurse	8 (1.2)
Physical therapist	122 (18.5)
Occupational therapist	507 (76.7)
Speech therapist	16 (2.4)
Workplace clinical phase	
Acute	219 (33.1)
Subacute	215 (32.5)
Chronic	227 (34.3)
Region of residence	
Alert region ^a^	506 (76.6)
Non-alert region	155 (23.4)
Clinical experience, years	
1–5	255 (38.6)
6–10	197 (29.8)
11–20	163 (24.7)
>20	46 (7.0)
Number of household members	
1 (participant only)	195 (29.5)
2 people	145 (21.9)
≥3	321 (48.6)
Children ≤ 18 years in household	
Yes	200 (30.3)
No	461 (69.7)
Financial situation	
No change	505 (76.4)
Worse than usual	66 (10.0)
Better than usual	90 (13.6)
Communication with family	
Same as usual	324 (49.0)
Less than usual	114 (17.2)
More than usual	223 (33.7)
Communication with friends	
Same as usual	192 (29.0)
Less than usual	403 (61.0)
More than usual	66 (10.0)

^a^ 13 prefectures were designated as alert regions.

**Table 2:** Mental health measurements according to gender, clinical workplace phase, and place of residence (*N* = 661).

		Gender			Workplace Phase				Place of Residence		
		Median (IQR)			Median (IQR)				Median (IQR)		
Measurements	Total	Men	Women	*p* Value	Acute	Subacute	Chronic	*p* Value	Alert Region ^a^	Non-Alert Region	*p* Value
GHQ-12	5 (3–7)	4 (2–7)	6 (3–7.75)	<0.001	5 (2–7)	5 (2–7)	6 (3–8)	0.85	5 (3–7)	5 (3–7)	0.79
Anxiety over COVID-19	9 (7–10)	8 (7–10)	9 (8–10)	<0.001	9 (7–10)	8 (7–10)	9 (7–10)	0.75	8 (7–10)	9 (7–10)	0.91
Health status	7 (5–9)	7 (5–9)	7 (5–8)	0.22	7 (5–9)	7 (5–9)	7 (4.5–8)	0.26	7 (5–8)	7 (5–9)	0.74
Satisfaction with daily life											
Satisfaction with leisure	2 (1–3)	3 (1–4)	2 (1–3)	<0.001	2 (1–3)	2 (1–3.5)	2 (1–4)	0.73	2 (1–3.5)	2 (1–3)	0.32
Satisfaction with work	6 (3–8)	6 (3–8)	5 (3.3–7)	0.22	5 (3–7)	6 (4–8)	5 (3–7)	0.01	5 (3.5–7)	6 (3–8)	0.59
Satisfaction with daily life activities	4 (3–7)	5 (3–7)	4 (3–7)	0.34	5 (3–7)	4 (3–6)	5 (3–7)	0.20	4 (3–7)	4 (3–7)	0.84
Satisfaction with new activities	6 (3–8)	6 (2–8)	6 (3–8)	0.25	6 (3–8)	6 (3–8)	6 (3–8)	0.73	6 (2–8)	6 (3–8)	0.45

Data were analyzed with the Mann–Whitney U test or Kruskal–Wallis H test. ^a^ 13 prefectures were designated as alert regions. Abbreviations: GHQ-12, General Health Questionnaire-12 (Japanese version); IQR, interquartile range.

**Table 3:** Characteristics and mental health measurements of the normal mental health and severe mental health problems groups.

Characteristics	Normal (*n* = 221), Number (%)	Severe (*n* = 440), Number (%)
Gender		
Men	130 (58.8)	177 (40.2)
Women	91 (41.2)	263 (59.8)
Age, years		
21–25	47 (21.3)	117 (26.6)
26–30	70 (31.7)	131 (29.8)
31–40	71 (32.1)	127 (28.9)
>40	33 (14.9)	65 (14.8)
Marital status		
Single	112 (50.7)	268 (60.9)
Married	109 (49.3)	172 (39.1)
Occupation		
Physician	5 (2.3)	3 (0.7)
Nurse	2 (0.9)	6 (1.4)
Physical therapist	50 (22.6)	72 (16.4)
Occupational therapist	160 (72.4)	347 (78.9)
Speech therapist	4 (1.8)	12 (2.7)
Workplace clinical phase		
Acute	74 (33.5)	145 (33.0)
Subacute	78 (35.3)	137 (31.1)
Chronic	69 (31.2)	158 (35.9)
Region of residence		
Alert region ^a^	170 (76.9)	336 (76.4)
Non-alert region	51 (23.1)	104 (23.6)
Clinical experience, years		
1–5	76 (34.4)	179 (40.7)
6–10	72 (32.6)	125 (29.4)
11–20	59 (26.7)	104 (23.6)
>20	14 (6.3)	32 (7.3)
Household members		
1	48 (21.7)	147 (33.4)
2	53 (24.0)	92 (20.9)
≥3	120 (54.3)	201 (45.7)
Children ≤ 18 years in household		
Yes	81 (36.7)	119 (27.0)
No	140 (63.3)	321 (73.0)
Financial situation		
No change	177 (80.1)	328 (74.5)
Worse than usual	18 (8.1)	48 (10.9)
Better than usual	26 (11.8)	64 (14.5)
Communication with family		
Same as usual	123 (55.7)	201 (45.7)
Less than usual	24 (10.9)	90 (20.5)
More than usual	74 (33.5)	149 (33.9)
Communication with friends		
Same as usual	95 (43.0)	97 (22.0)
Less than usual	101 (45.7)	302 (68.6)
More than usual	25 (11.3)	41 (9.3)
Measurements	Median (IQR)	Median (IQR)
Anxiety over COVID-19	8 (7–9)	9 (8–10)
Health condition	8 (7–9)	6 (4–8)
Satisfaction with daily life		
Satisfaction with leisure	3 (2–5)	2 (1–3)
Satisfaction with work	7 (5–8)	5 (3–7)
Satisfaction with daily life activities	6 (4–8)	4 (2–6)
Satisfaction with new activities	7 (4–8)	6 (2–7)

Normal mental health group: GHQ-12 score < 4. Severe mental health group: GHQ-12 score ≥ 4. ^a^ 13 prefectures were designated as alert regions. Abbreviations: GHQ-12, General Health Questionnaire-12; IQR, interquartile range.

**Table 4:** Risk factors associated with severe mental health problems ^a^.

			*p* Value	
Variables	No. with Mental Health Problems/Total No. (%)	Adjusted OR (95% CI)	Category	Overall
Characteristics				
Gender				
Men	177/307 (57.8)	1 (Reference)	NA	0.002
Women	263/354 (74.3)	1.83 (1.25–2.70)	0.002	
No. of household members				
1	147/195 (75.4)	1 (Reference)	NA	0.007
2	92/145 (63.4)	0.47 (0.27–0.80)	0.006	
≥3	201/321 (62.6)	0.52 (0.33–0.82)	0.005	
Communication with friends				
Same as usual	97/192 (50.5)	1 (Reference)	NA	<0.001
Less than usual	302/403 (74.9)	2.29 (1.51–3.46)	<0.001	
More than usual	41/66 (62.1)	1.29 (0.67–2.50)	0.45	
Measurements				
Anxiety over COVID-19	-	1.20 (1.08–1.34)	-	0.001
Health condition	-	0.76 (0.69–0.83)	-	<0.001
Satisfaction with work	-	0.82 (0.75–0.90)	-	<0.001
Satisfaction with new activities	-	0.92(0.86–0.99)	-	0.01

^a^ Defined as a General Health Questionnaire-12 score ≥ 4. This analysis was adjusted for health condition, satisfaction with leisure, satisfaction with work, satisfaction with daily life activities, satisfaction with new activities, communication with friends, communication with family, anxiety over COVID-19, place of residence, household members, children < 18 years in household, gender, age, marital status, workplace clinical phase, clinical experience, and economic situation, when appropriate. Category refers to the *p*-value for each category vs. the reference, while overall refers to the results of the logistic regression analysis. Abbreviations: CI, confidence interval; NA, not applicable; No., number; OR, odds ratio.

## Data Availability

The data presented in this study are available on request from the corresponding author.
